# Clinical guidelines contribute to the health inequities experienced by individuals with intellectual disabilities

**DOI:** 10.1186/1748-5908-7-42

**Published:** 2012-05-11

**Authors:** Lindsay AM Mizen, Marjorie L Macfie, Linda Findlay, Sally-Ann Cooper, Craig A Melville

**Affiliations:** 1 Psychiatry Training Scheme, South East Scotland Deanery, Edinburgh, Scotland, UK; 2 Learning Disabilities Psychiatry Higher Training Scheme, West of Scotland Deanery, Glasgow, Scotland, UK; 3 Department of Learning Disabilities Psychiatry, NHS Lanarkshire, Longdales, Kirklands, Bothwell, Scotland, UK; 4 Mental Health and Wellbeing, Institute of Health & Wellbeing, College of Medical, Veterinary and Life Sciences, University of Glasgow, Glasgow, Scotland, UK

**Keywords:** Intellectual disabilities, Clinical guidelines, Health inequalities

## Abstract

**Background:**

Clinical practice guidelines are developed to improve the quality of healthcare. However, clinical guidelines may contribute to health inequities experienced by disadvantaged groups. This study uses an equity lens developed by the International Clinical Epidemiology Network (INCLEN) to examine how well clinical guidelines address inequities experienced by individuals with intellectual disabilities.

**Methods:**

Nine health problems relevant to the health inequities experienced by persons with intellectual disabilities were selected. Clinical guidelines on these disorders were identified from across the world. The INCLEN equity lens was used as the basis for a purpose-designed, semistructured data collection tool. Two raters independently examined each guideline and completed the data collection tool. The data extracted by each rater were discussed at a research group consensus conference and agreement was reached on a final equity lens rating for each guideline.

**Results:**

Thirty-six guidelines were identified, one of which (2.8%) explicitly excluded persons with intellectual disabilities. Of the remaining 35, six (17.1%) met the first criterion of the equity lens, identifying persons with intellectual disabilities at high risk for the specific health problem. Eight guidelines (22.9%) contained any content on intellectual disabilities. Six guidelines addressed the fourth equity lens criterion, by giving specific consideration to the barriers to implementation of the guideline in disadvantaged populations. There were no guidelines that addressed the second, third, and fifth equity lens criteria.

**Conclusions:**

The equity lens is a useful tool to systematically examine whether clinical guidelines address the health needs and inequities experienced by disadvantaged groups. Clinical guidelines are likely to further widen the health inequities experienced by persons with intellectual disabilities, and other disadvantaged groups, by being preferentially advantageous to the general population. There is a need to systematically incorporate methods to consider disadvantaged population groups into the processes used to develop clinical guidelines.

## Introduction

International health policy is focused on tackling health inequalities [1]. However, the evidence suggests that public health interventions have not reduced inequalities [2], and the extent of health inequalities may be increasing [3,4].

The terms *inequalitiesdisparities*, and *inequities* have been used differently across geographical settings and specialities. Since the terms inequalities and disparities are used interchangeably, we will use inequalities in this paper. In this paper, we use the definitions of health inequalities and inequities provided by Whitehead [5]. Health inequalities refer to “measurable difference in health experience in health outcomes between different population groups – according to socio-economic status, geographical area, age, disability, gender or ethnic group.” Whitehead’s original definition of health equity has been adapted by the World Health Organisation (WHO) Commission on the Social Determinants of Health (CSDH), who define it as, “the absence of unfair and avoidable or medial differences on health amongst social groups” [6]. Health inequalities is the term most commonly used in intellectual disabilities research. Therefore, we will use the term inequalities when referring to previous intellectual disabilities research. Research and policy relevant to clinical guidelines most often refer to equity. Therefore, the terms equity and inequity will be used when referring to clinical guidelines, and thereafter in the Methods, Results, and Discussion sections of this manuscript.

Individuals with intellectual disabilities have consistently been reported to experience significant health inequalities [7-10]. Individuals with intellectual disabilities have been found in research studies to have increased mortality rates and reduced life expectancy [8], higher prevalence and incidence of specific physical [11] and mental health [12] needs, and poor access to evidence-based healthcare [13].

Clinical guidelines aim to improve the quality of healthcare and patient outcomes by systematically evaluating the evidence base and contributing to cost-effective healthcare [14]. Clinical guidelines have been proposed as one method to address inequalities in healthcare [15], and some national guideline organizations explicitly state in their policies that they consider health inequalities in the process used to select the topics for guideline development [16,17]. However, it has also been suggested that clinical guidelines increase the health inequities experienced by disadvantaged subgroups by differentially improving the health of people with an existing health advantage [8,18,19].

The aim of this study was to examine whether clinical guidelines address the health inequalities experienced by people with intellectual disabilities. Three research questions were examined:

1. Do clinical guidelines identify individuals with intellectual disabilities as a group who are at increased risk of the disorder?

2. Do clinical guidelines include content relevant to the specific health needs of persons with intellectual disabilities (*i.e.*, statements and recommendations)?

3. Do guideline development processes take into consideration the specific health needs of persons with intellectual disabilities?

## Methods

### Measurements

The International Clinical Epidemiology Network (INCLEN) supports the role that clinical guidelines can have in influencing practice and potentially reducing health inequities. To support groups developing clinical guidelines that address health inequities, INCLEN developed an “equity lens” to evaluate how well a clinical guideline addresses issues of equity [20]. This was one of 16 papers published by INCLEN as part of a program of work to advise the WHO Advisory Committee on Health Research on clinical guidelines. The five criteria of the equity lens and guidance on what to look for in clinical guidelines, as evidence for each of the five criteria [20], are shown in Table 1.

**Table 1:** **The INCLEN equity lens**[20]

**Equity lens criteria**	**What evidence for the criteria to look for in clinical guidelines**
1. Do the public health recommendations in the guidelines address a priority problem for disadvantaged populations?	Discussions on the burden of disease in disadvantaged populations.
2. Is there a reason to anticipate different effects of intervention in disadvantaged and privileged populations?	Discussions on differences between disadvantaged and privileged populations, in terms of biology of the disease, adherence, and baseline risks.
3. Are the effects of the intervention valued differently by disadvantaged compared to privileged populations?	Values may be assessed in guideline development panels through consultations with disadvantaged populations, involvement of their caregivers, reference to relevant research, or transparent reflection.
4. Is specific attention given to minimizing barriers to implementation in disadvantaged populations?	Discussion of barriers to implementation in disadvantaged populations and identification of strategies to overcome these barriers.
5. Do plans for assessing the impact of the recommendations include disadvantaged populations?	Plans for monitoring disadvantaged groups according to place of residence, race, occupation, gender, religion, education, socioeconomic status, or social network and capital.

INCLEN = International Clinical Epidemiology Network.

This study used the INCLEN equity lens to examine whether existing clinical guidelines address the health inequities experienced by people with intellectual disabilities. We also looked in more detail at the information provided on the guideline development process to identify potential means to address the health inequities experienced by individuals with intellectual disabilities and other disadvantaged groups.

To answer the research questions, the INCLEN equity lens was used to inform the design of a semistructured data collection tool, to extract information from the guidelines. Additional questions to look in more detail at the guideline development process and the content of guidelines were inserted. The data collection tool was divided into three sections:

1. Population relevance:

· Are any population groups, and specifically persons with intellectual disabilities, excluded from the guideline remit?

· Are groups at high risk of a disorder and, specifically, are persons with intellectual disabilities identified (equity lens criterion one)?

2. Guideline content:

· Are statements relevant to the specific health needs of individuals with intellectual disabilities made? Statements were divided into four categories: diagnosis and intervention (equity lens criteria two and three), guideline implementation (equity lens criteria four and five), and a category for other statements.

· Are recommendations specific to the health needs of individuals with intellectual disabilities made?

3. Guideline development process:

· Is an expert in intellectual disabilities included in the guideline development group?

· Is there evidence that the design of the literature search strategy could identify studies relevant to intellectual disabilities, for example, the use of relevant search terms or the citation of specific references relevant to intellectual disabilities?

· Is there evidence of consultation with people with intellectual disabilities and /or their carers in the development and implementation of the guideline (equity criterion three)?

### Selection of clinical guidelines

To meet the aim of the study, it was necessary that clinical guidelines on these disorders could meet at least one, or more, of the equity lens criteria. In the description of this first equity criterion [20], it is suggested that a problem is a priority for a disadvantaged group if there is an increased burden of disease and/or the prevalence of the disorder is higher in the disadvantaged group. To ensure that clinical guidelines could potentially address the inequities experienced by individuals with intellectual disabilities, we examined clinical guidelines on disorders known to have a higher prevalence in persons with intellectual disabilities. For example, it is known that there is research evidence from population-based epidemiological studies that obesity has a higher prevalence in individuals with intellectual disabilities. A clinical guideline on obesity could, therefore, potentially meet the first equity criterion with reference to intellectual disabilities.

On this basis, we selected nine health problems with research evidence from population-based, epidemiological studies demonstrating higher rates of these disorders in individuals with intellectual disabilities [11,13,21]. Intellectual disabilities’ psychiatry and psychology are established clinical specialties, internationally. Therefore, we considered that it may be more likely for clinical guidelines on mental health disorders to address the health inequities of individuals with intellectual disabilities. To take account of this, the list included disorders of physical and mental health. The health problems selected were obesity, osteoporosis, epilepsy, obstructive sleep apnea, upper gastrointestinal disorders, accidents/injuries or falls, bipolar affective disorder, dementia, and schizophrenia.

Since clinical guideline development is of relevance to health inequities globally, the inclusion of guidelines published in different countries was felt to be important. The National Guideline Clearing House lists guidelines produced worldwide so was used to identify a representative sample of relevant clinical guidelines (http://www.guideline.gov).

To improve the quality of clinical guidelines, organizations producing clinical guidelines are recommended to follow standardized procedures throughout the guideline development process. For example, there can be explicit requirements on the composition of the guideline development group or search strategies used. This standardized approach can be a potential strength of organizations with responsibility for the development of all clinical guidelines in a single country. Despite this standardization, we recognized that inclusion of a single guideline from a national organization could not be considered representative. On the other hand, no national organizations were identified as having published clinical guidelines on all nine health problems of relevance to this study. Therefore, to include guidelines of international relevance, while also attempting to include guidelines reflecting standardized procedures, it was decided to limit the sample to clinical guidelines published in English, from countries with guidelines available for two or more of the nine health problems selected (see Table 2).

**Table 2:** **Countries and organizations with clinical guidelines on the selected topics (see**** Additional file 1****)**

**Topic**	**Country**	
**Epilepsy**	UK	SIGN (original 2003, update 2005, review 2007)
		NICE (2004)
	Singapore	Ministry for Health (2007)
**Obesity**	UK	SIGN (2010)
		NICE (2006)
	Australia	National Health & Medical Research Council (2003)
	USA	National Heart, Lung & Blood Institute (1998)
	Canada	Canadian Medical Association (2007)
	Malaysia	Ministry for Health (2004)
**Osteoporosis**	UK	SIGN (original 2003, update 2004, review 2007)
	USA	American College of Physicians (2008)
	Canada	Society of Obstetricians & Gynaecologists of Canada (2009)
	Singapore	Singapore Ministry of Health (2009)
	Malaysia	Malaysian Ministry of Health (2003)
**Dementia**	UK	SIGN (original 2006, review 2009)
		NICE (2007)
	NZ	Ministry of Health (1997)
	USA	American Psychiatric Association (2007)
	Canada	Canadian Medical Association (2008)
	Singapore	Ministry of Health (2007)
**Schizophrenia**	UK	SIGN (original 1998, review 2005)
		NICE (2009)
	NZ	Royal Australian & New Zealand College of Psychiatrists (2004)
	Australia	same as New Zealand
	Canada	Canadian Psychiatric Association (2005)
**Upper gastrointestinal problems**	UK	SIGN (original 2003, review 2009)
		NICE (2004)
	NZ	New Zealand Guidelines Group (2004)
	Canada	Canadian Medical Association (June 2000, update Sept 2000)
**Accidents/injuries & falls**	UK	NICE (2004)
	Canada	Canadian Task Force on Preventive Health Care (June 2003)
**Bipolar disorder**	UK	SIGN (original 2005, review 2009)
		NICE (2006)
	NZ	Royal Australian & New Zealand College of Psychiatrists (2004)
	Australia	same as New Zealand
	Canada	Canadian Network for Mood & Anxiety Treatments (2005, update 2007)
**Obstructive sleep apnea**	UK	SIGN **(**original 2003, review 2009)
	USA	American Academy of Sleep Medicine 2006

SIGN = Scottish Intercollegiate Guideline Network; NICE = National Institute for Health and Clinical Excellence.

### Data extraction process

All of the guidelines were independently searched electronically by two medically qualified doctors (LAMM and MLM) to identify any content of specific relevance to intellectual disabilities. Since there are several different terms for intellectual disabilities used internationally, individual search terms used to identify relevant content included *disability*, *disabilities*, *retardation*, *impairment*, *handicap*, *intellectual*, *learning*, and *mental*. The two doctors independently completed the data extraction tool for each guideline. The data sets produced by the independent raters were systematically discussed at a consensus meeting of the research team. If there were any discrepancies between raters, the section of the guideline in question was re-examined by the research team to reach consensus agreement for each guideline.

In extracting data relevant to the first equity lens criterion, we wanted to examine whether guidelines did not see the identification of high-risk groups as part of the guideline remit or whether they were including information on some high-risk groups and omitting intellectual disabilities. To achieve this, the raters scored guidelines on whether the guideline identified any population group at high risk of the health problem, and then separately scored whether the guideline identified individuals with intellectual disabilities at high risk.

## Results

The 36 guidelines in Table 2, from seven countries, met the inclusion criteria for the study ( Additional file1).

One of the guidelines (2.8%) explicitly excluded persons with intellectual disabilities. Of the remaining 35, six (17.1%) met the first criterion of the equity lens, identifying persons with intellectual disabilities at high risk for the specific health problem.

### Equity lens criteria

As described above, the study design meant that all 36 guidelines could potentially meet the first equity lens criteria for intellectual disabilities. Although 29 guidelines (82.9%) met the first equity lens criteria by identifying population subgroups at increased risk for this specific disorder, only six of these (71.1%) identified people with intellectual disabilities at increased risk of the disorder (Table 3). Therefore, although the majority of guidelines met this criterion to some extent, relatively few did so with regard to intellectual disabilities. The section examining the guideline development process (see below) suggests possible reasons for the inconsistency with which guidelines meet the first equity lens criteria for disadvantaged groups.

**Table 3:** Guidelines meeting equity lens criteria or containing additional content on intellectual disabilities

	**Equity lens criterion 1**		**Equity lens criterion 4 (number of comments)**	**Non-lens ID statement categories (number of comments)**		
	**Any groups identified at increased risk**	**ID identified at increased risk**	**Barriers to implementation**	**Diagnosis**	**Intervention**	**Other**
Obesity	SIGN	NICE	NICE (5)	SIGN (1)	NICE (6)	NICE (4)
	NICE	SIGN			SIGN (1)	SIGN (2)
	Australia					
	USA					
	Canada					
	Malaysia					
Osteoporosis	SIGN		None	None	None	None
	USA					
	Canada					
	Singapore					
**Epilepsy**	NICE	SIGN	NICE (5)	NICE (18)	NICE (12)	NICE (19)
	SIGN	NICE	SIGN (9)	SIGN (3)	SIGN (6)	SIGN (5)
**Obstructive sleep apnea**	SIGN		None	None	None	None
	USA					
**Upper gastrointestinal problems**	NICE		None	None	None	None
	New Zealand					
	Canada					
**Accidents/injuries, falls**	NICE		None	None	None	None
	Canada					
**Dementia**	NICE	NICE	NICE (10)	NICE (9)	NICE (9)	NICE (4)
	USA					
	Canada					
	Singapore					
	Malaysia					
**Schizophrenia**	New Zealand/Australia		None	Canada (1)	None	None
**Bipolar affective disorder**	SIGN	NICE	NICE (1)	NICE (1)	None	NICE (1)
	NICE			SIGN (1)		SIGN (1)
	Canada					

ID = intellectual disabilities; SIGN = Scottish Intercollegiate Guideline Network; NICE = National Institute for Health and Clinical Excellence.

Five guidelines (shown in Table 3) made some statement about the barriers to implementation of the guideline for persons with intellectual disabilities and were rated as meeting equity lens criterion number four. The potential barriers to implementation recognized in the guidelines included the need to consider the decision-making capacity of health service users, problems individuals with intellectual disabilities experience accessing generic healthcare services, and challenges faced when service users make the transition from children to adult’s services.

None of the guidelines were rated as addressing equity lens criteria two, three, or five.

### Guideline content

In order to look beyond the equity lens criteria, we examined the 35 guidelines for all content relevant to specific needs of people with intellectual disabilities. Eight guidelines (22.9%) contained any content making specific reference to intellectual disabilities. Of these eight, seven (20%) contained statements on diagnosis (*e.g.*, descriptions of different presentations in intellectual disabilities’ populations, specific diagnostic challenges), five (14.3%) included content relevant to interventions ( *e.g.*, the need to develop care plans accommodating the needs of individuals with intellectual disabilities), and seven (20%) included other relevant content ( *e.g.*, making reference to the lack of evidence available for the intellectual disabilities population). The Scottish Intercollegiate Guideline Network (SIGN) obesity guideline included an appendix listing useful resources for professionals working with individuals with intellectual disabilities or communication difficulties.

Specific intellectual disabilities recommendations were made in six guidelines (17.1%). As shown in Table 4, none of these were evidence-based recommendations, and most often, the recommendations emphasize the need for appropriate specialist referral.

**Table 4:** Guidelines with recommendations specific to persons with intellectual disabilities

**Theme**	**Guidelines**	**Specific examples of recommendations**
Specialist management	NICE Obesity	People with learning disabilities and those supporting them should have access to specialist advice and support regarding dementia. (NICE Dementia)
	NICE Epilepsy	
	SIGN Epilepsy	
	NICE Dementia	
Communication/consultation	NICE Obesity	Information in an accessible form should be available to clients and carers. (SIGN Epilepsy)
	SIGN Epilepsy	
Equality	NICE Epilepsy	Every therapeutic option should be explored in individuals with epilepsy in the presence or absence of learning disabilities. (NICE Epilepsy)
	NICE Dementia	
	NICE Bipolar	
Service/training issues	NICE Dementia	Health and social care staff working in care environments where younger people are at risk of developing dementia, such as those catering to people with learning disabilities, should be trained in dementia awareness. (NICE Dementia)
Diagnostic issues	NICE Epilepsy	Patients with mental retardation, especially those who are nonverbal, may be more challenging to assess; collateral information from caregivers is important. Cognitive and functional testing to delineate the patient’s developmental level and relative strengths and weaknesses are also essential. (Canada Schizophrenia)
	NICE Dementia	
	Canada Schizophrenia	
Treatment issues	NICE Epilepsy	In making a management plan for an individual with learning disabilities and epilepsy, particular attention should be paid to the possibility of adverse cognitive and behavioral effects of anti-epileptic drug therapy. (NICE Epilepsy)
	SIGN Epilepsy	

SIGN = Scottish Intercollegiate Guideline Network; NICE = National Institute for Health and Clinical Excellence.

### Guideline development process

Four (11.4%) of the 35 guideline development groups included members with intellectual disabilities expertise. An intellectual disabilities dietician was on the specialist review panel for the SIGN obesity guideline but was not part of the development group.

This lack of expertise in the development guideline groups may, in part, explain the small number of guidelines identifying individuals with intellectual disabilities at high risk (equity criterion one) or making specific reference to intellectual disabilities anywhere in the guideline. This effect could have been compounded by the fact that only two (5.7%) of the guidelines contained evidence that the literature search strategy had included search terms that would have identified evidence relevant to the health needs of individuals with intellectual disabilities (NICE obesity and NICE epilepsy). Although several guidelines that did not use specific intellectual disabilities search terms included references relevant to intellectual disabilities in the bibliography (NICE obesity, NICE epilepsy, SIGN epilepsy, NICE dementia, and NICE bipolar affective disorder), it could be that the methodology used to identify relevant evidence does not readily identify intellectual disabilities research. One final point of note from the examination of the guideline development process is that only one guideline (SIGN epilepsy) carried out consultations with service users and service users with intellectual disabilities and carers.

## Discussion

This is the first study to examine whether clinical guidelines address the health inequities experienced by individuals with intellectual disabilities. We found that the majority of clinical guidelines did not contain content relevant to intellectual disabilities or meet any of the equity lens criteria for intellectual disabilities. Since there is available evidence to allow all the guidelines to meet equity lens criterion one, the findings of this study suggest that current guideline development processes may be missing an important opportunity to address the inequities experienced by individuals with intellectual disabilities. For example, none of the guidelines on osteoporosis, obstructive sleep apnea, upper gastrointestinal problems, and accidents/injuries or falls identified individuals with intellectual disabilities as being at increased risk. If this had been included in the guidelines, it could have led to better assessment and diagnosis of those health problems that individuals with intellectual disabilities are at high risk of experiencing. Appropriate diagnosis and management would subsequently be expected to reduce the inequities experienced by persons with intellectual disabilities by allowing fairer access to relevant management and services.

### Strengths and limitations

This study benefited from the use of the INCLEN equity lens, developed by a group of international experts, independent of the research group involved in this study. We found that the five equity lens criteria provided a useful framework to consider whether clinical guidelines address the inequities experienced by individuals with intellectual disabilities. The use of the equity lens as the basis of the study ensures that the process can be repeated in future intellectual disabilities studies and studies examining the relevance of clinical guidelines to other disadvantaged groups.

The process used to rate the clinical guidelines was a strength of this study. Three separate aspects of the process reduced the risk of rater error: independent rating of the guidelines by two researchers, use of a standardized data extraction tool, and a consensus group process to agree on final ratings for each guideline. To further improve the rigor of the rating process, any future studies could consider blinding the researchers and consensus process to which guideline was being rated.

Since clinical guidelines are of international significance to the inequities experienced by individuals with intellectual disabilities, the inclusion of guidelines from different countries adds to the relevance of the findings. The development of guidelines is highly complex and influenced by multiple factors at any one time. A potential weakness of the study is that the research group has little experience of the complexities surrounding the guideline development process used across the organizations that produced the guidelines included in this study. The study is exploratory in nature and will hopefully allow equity to be considered further as part of guideline development. However, future studies of this nature would benefit from involvement of international experts in clinical guideline development.

### Improving the equity of clinical guidelines for intellectual disabilities

Several of the guidelines reviewed illustrate that it is possible to improve the equity of clinical guidelines in relation to intellectual disabilities. For example, five guidelines made statements on the barriers to implementation (Table 3). This suggests that there are feasible changes that can be made to the clinical guideline development process to address inequities.

INCLEN used the PROGRESS framework shown in Table 5[22] to identify disadvantaged groups that the guideline equity lens should be focused on. The PROGRESS framework has been used to examine the equity of systematic reviews [23]. Subsequently, PROGRESS-Plus (Table 5) has been proposed as a more comprehensive listing of population subgroups at risk of experiencing health inequities [24]. Therefore, guideline development groups could use the PROGRESS-Plus framework as part of the processes used to produce clinical guidelines. Given the exploratory nature of this study and the complexity of developing guidelines, it is not our intention to be prescriptive about how organizations make use of the PROGRESS-Plus framework. Any decisions on changes in the guideline development process can only be made in the context of national policies and priorities. For the purpose of illustration, one example would be for national guideline organizations to maintain a list of individuals with expert knowledge on intellectual disabilities. Prior to the start of the guideline development process, the guideline organization could seek the opinion of a relevant expert on whether there is potential for the guideline on a specific topic to address inequities. This would then allow the guideline organization to consider the feasibility of adjusting the guideline development process to meet the equity lens criteria.

**Table 5:** The PROGRESS framework and additional items included in PROGRESS-Plus

**PROGRESS **[22]	**Plus **[24]
**P**lace of residence	Age
**R**ace	Disability
**O**ccupation	Sexual orientation
**G**ender	Other vulnerable groups
**R**eligion	
**E**ducation	
**S**ocioeconomic status	
**S**ocial network and capital	

Previous studies that examined the equity of guidelines regarding gender [25] and ethnicity [26] made suggestions on how a guideline development process can be adapted to consider the needs of these specific groups. The majority of these considerations could also be used for other groups listed in the PROGRESS-Plus framework, including people with intellectual disabilities. In Table 6, we suggest reasonable adjustments that organizations can make to the guideline development process in order to improve the equity of guidelines in relation to intellectual disabilities. As stated above, we lack the necessary expertise to make statements on necessary changes that organizations should make, internationally. Instead, the suggestions in Table 6 are provided as examples of changes that could improve the equity of guidelines for individuals with intellectual disabilities. We recognize that such changes have resource implications and organizations are best placed to consider the feasibility of amendments to the guideline development process.

**Table 6:** Reasonable adjustments to support guideline development processes to improve the equity of clinical guidelines for individuals with intellectual disabilities

**1.**	**Clinical guideline organizations**
a)	Develop an organizational *statement* that all guideline development groups will specifically consider intellectual disabilities and make reasonable adjustments to improve guideline equity
b)	Introduce a procedure for a relevant expert on intellectual disabilities to review all topics selected for guideline development and, where appropriate, advise the development group
c)	Develop a literature search strategy relevant to intellectual disabilities for use along with topic-specific search strategies
**2.**	**Clinical guideline development groups**
a)	Where appropriate, make use of the intellectual disabilities literature search to consider how the clinical guideline can address those equity lens criterion of relevance
b)	In formulating recommendations, consider how applicable they are to the health of individuals with intellectual disabilities, and suggest reasonable adjustments in appendices on the equity of guideline implementation
c)	Invite stakeholders relevant to intellectual disabilities to participate in the consultation process on draft versions of guidelines
d)	Publish easy-to-read versions for service users, in parallel with the full guideline

The potential solutions shown in Table 6, or suggested in previous studies [25,26], would have a greater impact if they were taken forward by the international collaborations that exist to influence the rigor and quality of published clinical guidelines, such as the Guideline International Network (G-I-N; http://www.g-i-n.net ) or the Appraisal of Guidelines Research and Evaluation Enterprise (AGREE; http://www.agreetrust.org). Perhaps the first stage would be to ensure that information about the work on guideline equity by INCLEN is disseminated. A revised AGREE II instrument has been published [27] and does not include any questions to help individuals appraising guidelines to examine whether the guidelines address, or make worse, issues of health inequity [28]. One suggestion is that future work to revise the AGREE instrument could incorporate issues around improving guideline equity.

The National Guideline Clearing house website (http://www.guideline.gov) does not make reference to any guidelines specifically dedicated to the health needs and priorities of individuals with intellectual disabilities. Hence, it is important that more generic guidelines do address the health needs of people with intellectual disabilities where possible. Findings from this study suggest that, at present, clinical guidelines may be missing the opportunity to have a positive impact on the health inequities experienced by person with intellectual disabilities, and may even be making these worse. Content of clinical guidelines should endeavor to meet the five INCLEN equity lens criteria for individuals with intellectual disabilities and other groups. To achieve this, guidelines should recognize different disease patterns, including different prevalence, incidence, and etiologies; the interventions and supports needed by disadvantaged groups; and any necessary reasonable adjustments to healthcare delivery, organization, and policy in order to equally meet the needs of individuals with intellectual disabilities. For example, recommendations of clinical guidelines for the investigation of upper gastrointestinal cancers—which are more common in individuals with intellectual disabilities [29]—have little relevance for individuals with intellectual disabilities who do not have verbal communication skills if they require self-report of dyspepsia. This highlights the fundamental importance of clinical guidelines addressing inequities experienced by individuals with intellectual disabilities, recognizing the need to necessarily engage carers, as well as the person with intellectual disabilities in assessments and interventions.

## Conclusion

Many of the issues raised with reference to intellectual disabilities are also relevant to other disadvantaged groups within the PROGRESS-Plus framework and should be considered by clinical guideline development groups.

Most clinical guidelines do not yet meet the published equity lens criteria and may, therefore, be contributing to the health inequities experienced by individuals with intellectual disabilities and other disadvantaged groups. Therefore, contrary to the specified purpose of clinical guidelines, the resources devoted to clinical guideline development internationally may result in a widening of existing health inequities. Reasonable adjustments could be adopted to address this, so that the guidelines become part of a broader strategy to reduce health inequities.

## Competing interests

CAM is a member of the SIGN Mental Health Learning Disabilities Subgroup. None of the other authors have any competing interests.

## Authors’ contributions

CAM conceived and designed the work, with contributions from SAC and LF. LAMM and MLM and contributed to the literature review and writing and acquired the guideline data, with oversight from CAM and SAC. CAM interpreted the guideline data with contributions from SAC and LF. All authors read and approved the final manuscript.

## Supplementary Material

Additional file 1 **Details of clinical guidelines and websites for download of clinical guidelines (all guidelines accessed 21/07/2011).**Bibliography for guidelines used in the study and URLs for online access to guidelines. Click here for file
