# Supplementary material for: Clinical guidelines contribute to the health inequities experienced by individuals with intellectual disabilities
Source: Implement Sci. 2012 May 11;7:42. doi: 10.1186/1748-5908-7-42 (PMC3479008; doi:10.1186/1748-5908-7-42)
Supplement: Additional file 1 — Details of clinical guidelines and websites for download of clinical guidelines (all guidelines accessed 21/07/2011).Bibliography for guidelines used in the study and URLs for online access to guidelines. [file 1748-5908-7-42-S1.doc]

**Details of clinical guidelines and websites for download of clinical guidelines (all guidelines accessed 23/02/2012)**

**National Institute for Health and Clinical Excellence (NICE) guidelines**

Stokes T, Shaw EJ, Juarez-Garcia A, Camosso-Stefinovic J, Baker R. Clinical Guidelines and Evidence Review for the Epilepsies: diagnosis and management in adults and children in primary and secondary care. (http://www.nice.org.uk/nicemedia/pdf/CG020fullguideline.pdf) London: Royal College of General Practitioners, 2004.

National Institute for Health and Clinical Excellence. Obesity: the prevention, identification, assessment and management of overweight and obesity in adults and children. (http://guidance.nice.org.uk/index.jsp?action=folder&o=30362) London, National Institute for Health and Clinical Excellence, 2006.

National Collaborating Centre for Mental Health. A NICE-SCIE guideline of supporting people with dementia and their carers in health and social care. (http://www.nice.org.uk/nicemedia/pdf/CG42Dementiafinal.pdf) London, British Psychological Society and Royal College of Psychiatrists, 2006.

National Collaborating Centre for Mental Health. Core interventions in the treatment and management of schizophrenia in primary and secondary care. (http://www.nice.org.uk/nicemedia/pdf/CG82FullGuideline.pdf) London, National Institute for Health and Clinical Excellence, 2009.

North of England Dyspepsia Guideline Development Group. Dyspepsia: managing dyspepsia in adults in primary care. (http://www.nice.org.uk/nicemedia/pdf/CG017fullguideline.pdf) Newcastle, University of Newcastle, 2004.

National Collaborating Centre for Mental Health. The management of bipolar disorder in adults, children and adolescents, in primary and secondary care. (http://www.nice.org.uk/nicemedia/pdf/CG38fullguideline.pdf) London, British Psychological Society and Royal College of Psychiatrists, 2006.

National Collaborating Centre for Nursing and Supportive Care. Clinical practice guideline for the assessment and prevention of falls in older people. (http://www.nice.org.uk/nicemedia/pdf/CG021fullguideline.pdf) London, Royal College of Nursing, 2004.

**Scottish Intercollegiate Guideline Network (SIGN) guidelines**

Scottish Intercollegiate Guideline Network. Diagnosis and management of epilepsy in adults. (http://www.sign.ac.uk/pdf/sign70.pdf; Review report http://www.sign.ac.uk/pdf/2007epilepsyadult.pdf) Edinburgh, Scottish Intercollegiate Guideline Network, 2005.

Scottish Intercollegiate Guideline Network. Obesity in Scotland. Integrating prevention with weight management. (http://www.sign.ac.uk/pdf/sign8.pdf; Review report http://www.sign.ac.uk/pdf/2005obesityreport.pdf) Edinburgh, Scottish Intercollegiate Guideline Network, 1996.

Scottish Intercollegiate Guideline Network. Management of osteoporosis. (http://www.sign.ac.uk/pdf/sign71.pdf ; Review report http://www.sign.ac.uk/pdf/2007osteoporosis.pdf) Edinburgh, Scottish Intercollegiate Guideline Network, 2003.

Scottish Intercollegiate Guideline Network. Management of patients with dementia. (http://www.sign.ac.uk/pdf/sign86.pdf) Edinburgh, Scottish Intercollegiate Guideline Network, 2006.

Scottish Intercollegiate Guideline Network. Psychosocial interventions in the management of schizophrenia. (http://www.sign.ac.uk/pdf/sign30.pdf: Review report http://www.sign.ac.uk/pdf/2005schizophreniareport.pdf) Edinburgh, Scottish Intercollegiate Guideline Network, 1998.

Scottish Intercollegiate Guideline Network. Dyspepsia. (http://www.sign.ac.uk/pdf/sign68.pdf; Review report http://www.sign.ac.uk/pdf/2007dyspepsia.pdf) Edinburgh, Scottish Intercollegiate Guideline Network, 2003.

Scottish Intercollegiate Guideline Network. Bipolar affective disorder. (http://www.sign.ac.uk/pdf/sign82.pdf) Edinburgh, Scottish Intercollegiate Guideline Network, 2005.

Scottish Intercollegiate Guideline Network. Management of obstructive sleep apnoea/ hypopnoea in adults. (http://www.sign.ac.uk/pdf/sign73.pdf; Review report http://www.sign.ac.uk/pdf/2007sleep.pdf) Edinburgh, Scottish Intercollegiate Guideline Network, 2003.

**Guidelines from New Zealand**

Ministry of Health. Guidelines for the support and management of people with dementia. (http://www.nzgg.org.nz/guidelines/0045/Guidelines_For_People_With_Dementia.pdf) Wellington, Ministry of Health, 1997.

Royal Australian and New Zealand College of Psychiatrists Guidelines Team for the Treatment of Schizophrenia and Related Disorders. Clinical practice guidelines for the treatment of schizophrenia and related disorders. (http://www.ranzcp.org/images/stories/ranzcpattachments/Resources/Publications/CPG/Clinician/CPG_Clinician_Full_Schizophrenia.pdf) *Australian and New Zealand Journal of Psychiatry* 2005; **39**: 1-30.

New Zealand Guidelines Group. Management of dyspepsia and heartburn. (http://www.nzgg.org.nz/guidelines/0077/Dyspepsia_Guideline_(web).pdf) Wellington, New Zealand Guidelines Group, 2004.

Royal Australian and New Zealand College of Psychiatrists Guidelines Team for Bipolar Disorder. Clinical practice guidelines for the treatment of bipolar disorder. (http://www.ranzcp.org/images/stories/ranzcp-attachments/Resources/Publications/CPG/Clinician/CPG_Clinician_Full_Bipolar.pdf) *Australian and New Zealand Journal of Psychiatry* 2004; **38**: 280-305.

**Guidelines from Australia**

National Health and Medical Research Council. Clinical Practice guidelines for the management of overweight and obesity in adults. (http://www.health.gov.au/internet/main/publishing.nsf/Content/obesityguidelines-guidelines-adults.htm/$FILE/adults.pdf)

Canberra, National Health and Medical Research Council, 2003.

**USA guidelines**

National Heart, Lung & Blood Institute. Clinical guidelines on the identification, evaluation and treatment of overweight and obesity in adults. (http://www.nhlbi.nih.gov/guidelines/obesity/ob_gdlns.pdf) Bethesda, National Institutes of Health, 1998.

Qaseem, A., Snow, V.,Shekelle, P., Hopkins, R., Forciea, M.A., Owens, D.K. Pharmacologic. Treatment of Low Bone Density or Osteoporosis to Prevent Fractures: A Clinical Practice Guideline from the American College of Physicians (http://www.annals.org/cgi/reprint/149/6/404.pdf) *Annals of Internal Medicine* 2008;**149**:404-415.

American Psychiatric Association. Practice guideline for the treatment of patients with Alzheimer’s disease and other dementias. (http://www.psychiatryonline.com/pracGuide/loadGuidelinePdf.aspx?file=AlzPG101007) Arlington, American Psychiatric Association, 2007.

American Psychiatric Association. Practice guideline for the treatment of patients with bipolar disorder. (http://www.psychiatryonline.com/pracGuide/loadGuidelinePdf.aspx?file=Bipolar2ePG_05-15-06; Guideline review http://www.psychiatryonline.com/content.aspx?aID=148434) Arlington, American Psychiatric Association, 2002.

Morgenthaler TI; Kapen S; Lee-Chiong T et al. Practice parameters for the medical therapy of obstructive sleep apnea. (http://www.aasmnet.org/Resources/PracticeParameters/PP_MedicalTherapyOSA.pdf) *Sleep* 2006; **29**(8):1031-1035.

**Canadian guidelines**

Lau, D. C.W., Douketis, J.D., Morrison, K.M., Hramiak, I.M., Sharma, A. M., Ur, E Canadian clinical practice guidelines for the management and prevention of obesity in adults and children. (http://www.cmaj.ca/cgi/data/176/8/S1/DC1/1) *Canadian Medical Association Journal* 2007; **176** (8): Online 1-117.

Society of Obstetricians and Gynaecologists of Canada. Clinical practice guideline: menopause and osteoporosis*.* (http://www.sogc.org/guidelines/documents/Menopause_JOGC-Jan_09.pdf) *Journal of Obstetrics and Gynaecology* 2009; **31 (suppl 1)**: S1-52.

Hermann, N., Gauthier, S. Diagnosis and treatment of dementia: management of severe Alzheimer disease. (http://www.cma.ca/index.cfm/ci_id/54343/la_id/1.htm?orgId=911210&orgName=Third%20Canadian%20Consensus%20Conference%20on%20the%20Diagnosis%20and%20Treatment%20of%20Dementia) *Canadian Medical Association Journal* 2008; **179**: 1278-1287.

Canadian Psychiatric Association. Clinical practice guidelines: treatment of schizophrenia. (https://ww1.cpa-apc.org/Publications/Clinical_Guidelines/schizophrenia/november2005/cjp-cpg-suppl1-05_full_spread.pdf)

*Canadian Journal of Psychiatry* 2005; **50** **(suppl 1):** S1-57.

Canadian Dyspepsia Working Group An evidence-based approach to the management and investigation of dyspepsia in the era of *Helicobacter pylori*. (http://www.cmaj.ca/cgi/reprint/162/12_suppl/s3 Update http://www.cmaj.ca/cgi/reprint/163/6/696)

*Medical Association Journal 2000*; **162 (suppl. 12)**: S1-23.

Norris MA, Walton RE, Patterson CJS, Feightner JW. and the Canadian Task Force on Preventive Health Care. Prevention of Falls in Long- Term Care Facilities: Systematic Review and Recommendations. CTFPHC Technical Report. London, ON:

Canadian Task Force, 2003.

Yatham LN, Kennedy SH, O’Donovan C, et al. Canadian Network for Mood and Anxiety Treatments (CANMAT) guidelines for the management of patients with bipolar disorder: consensus and controversies. (http://www.canmat.org/resources/PDF/Bipolar_Guidelines.pdf; Update http://www.canmat.org/resources/PDF/Bipolar_Guidelines_update_2006.pdf) *Bipolar Disorders* 2005: **7 (suppl. 3)**: S5–69.

**Guidelines from the Ministry of Health, Singapore**

Ministry of Health. Clinical practice guidelines: epilepsy in adults. (http://www.moh.gov.sg/mohcorp/uploadedFiles/Publications/Guidelines/Clinical_Practice_Guidelines/Diagnosis%20and%20Management%20of%20epilepsy%20in%20adults.pdf f%20epilepsy%20in%20adults.pdf) Singapore, Ministry of Health, 2007.

Ministry of Health. Osteoporosis. (http://www.moh.gov.sg/mohcorp/uploadedFiles/Publications/Guidelines/Clinical_Practice_Guidelines/cpg_Osteoporosis%20-%20Jan%202009.pdf) Singapore, Ministry of Health, 2009.

Ministry of Health. Clinical practice guidelines: dementia. (http://www.moh.gov.sg/mohcorp/uploadedFiles/Publications/Guidelines/Clinical_Practice_Guidelines/Dementia.pdf)

Singapore, Ministry of Health, 2007.

Ministry of Health. Management of Helicobacter Pylori infection. (http://www.moh.gov.sg/mohcorp/uploadedFiles/Publications/Guidelines/Clinical_Practice_Guidelines/cpg_Management_of_Helicobacter_pylori_Infection-Sep_2004.pdf) Singapore, Ministry of Health, 2004.

**Guidelines from the Ministry of Health, Malaysia**

Ministry of Health. Clinical practice guidelines on management of obesity. (http://www.moh.gov.my/MohPortal/cpgDetail.jsp?action=view&id=20 Putrajaya, Ministry of Health, 2004.

Ministry of Health. Clinical practice guidelines on management of dementia. (http://www.moh.gov.my/MohPortal/cpgDetail.jsp?action=view&id=8) Putrajaya, Ministry of Health, 2003.
